# Risk Factors for Acute Respiratory Infections in Children Between 0 and 23 Months of Age in a Peri-Urban District in Pakistan: A Matched Case–Control Study

**DOI:** 10.3389/fped.2021.704545

**Published:** 2022-01-10

**Authors:** Fatima Mir, Shabina Ariff, Maria Bhura, Suhail Chanar, Apsara Ali Nathwani, Muhammad Jawwad, Amjad Hussain, Arjumand Rizvi, Muhammad Umer, Zahid Memon, Atif Habib, Sajid Bashir Soofi, Zulfiqar A. Bhutta

**Affiliations:** Department of Pediatrics and Child Health, The Aga Khan University, Karachi, Pakistan

**Keywords:** acute respiratory infection, risk factors, children, peri-urban setting, Pakistan

## Abstract

**Background:** Acute respiratory infection (ARI) accounts for nearly 15% of all childhood mortality in South Asia, with children from rural areas at higher risk due to inaccessibility to healthcare facilities. We therefore aimed to identify risk factors associated with ARI in children under 2 years of age in rural Pakistan.

**Methods:** A retrospective 1:2 matched case–control study was conducted between October and December 2018 in Taluka Kotri, Jamshoro District of Pakistan. Cases were identified as children between 0 and 23 months of age with a history of fever, cough, sore throat, fast breathing, difficulty breathing, or chest indrawing in the 2 weeks prior to the survey. Controls were participants without symptoms of ARI, matched based on age in months. Data analysis was conducted using STATA version 15. Univariate and multivariable conditional logistic regression analyses were used to identify factors associated with ARI, and *p* < 0.05 was considered statistically significant.

**Results:** We identified 1,071 cases of ARI who were matched with 2,142 controls. Multivariable analysis revealed that female gender [odds ratio (OR) 0.78, 95% confidence interval (CI): 0.67–0.91], exclusive breastfeeding (OR 0.81, 95% CI: 0.69–0.97), and comorbidity with diarrhea (OR: 1.64, 95% CI: 1.40–1.91) were significantly associated with ARI.

**Conclusion:** Pakistan continues to progress toward reducing childhood mortality, particularly ARI-related deaths, for which it bears a great burden. This study identifies risk factors such as the male gender, breastfeeding, and comorbidities with diarrhea, which could open grounds for further programmatic implications in targeting a multifaceted approach to reducing incidences of ARI in rural areas of the country.

## Background

Acute respiratory infections (ARIs) are one of the leading causes of childhood morbidity and mortality worldwide, contributing to a third of the under-five deaths in lower income countries (1, 2). Acute respiratory infections include both upper and lower respiratory tract infections, with the common cold and influenza being the most common ARIs (3). Symptoms of ARI consist of short, rapid breathing, or difficulty breathing that is chest related. Pneumonia is a presentation of ARI and is solely responsible for 15% of global childhood deaths across the world (2). Symptoms presented with pneumonia include fast breathing and chest indrawing (4). The Global Burden of Disease (2019) study reports that lower respiratory tract infections are the second highest cause of burden in children (5). As of 2015, pneumonia kills 0.9 million children under five every year and is responsible for 15% of under-five deaths in South Asia (6).

Pakistan has a childhood mortality of 67 deaths per 1,000 live births (2). The country bears the third highest burden of global pneumonia deaths in children under-five, at 640,000 deaths per year (6). The incidence of ARI in under-five children has declined from 16% in 2012–2013 to 14% in 2017–2018, with the highest prevalence of symptoms among children between 6 and 23 months of age (7). Another study has reported a prevalence of pneumonia in children under five at 29% in Swat Valley, Pakistan (8). The study also reported a higher incidence of ARI among the lower social class, overcrowded houses, and houses that burnt fuel for cooking (8). According to the Multiple Indicator Cluster Survey (MICS) Punjab (2017–2018), “tackling leading killer diseases like pneumonia” is a direction toward attaining Sustainable Development Goal 3.2 by 2030, which aims to reduce childhood mortality to 25 deaths per 1,000 live births (9, 10).

Although pneumonia is diagnosed with chest x-rays, sputum cultures, and blood tests, poor infrastructure and lack of access to diagnostics in lower income countries result in diagnosis by symptom presentation (6). This situation is exacerbated in rural and poorer settings, where access to healthcare and availability of health facilities is scarce, thereby preventing timely management of respiratory infections in children. Progress has been made to manage disease through oxygen therapy intervention, but barriers continue to exist at every level, particularly in rural and poor areas, where health-seeking behavior is the least common (6, 7). Moreover, younger children are at higher risk to developing ARI due to their ongoing lung development, increased exposure to infection, and lower immunity (11, 12).

Despite a decrease in mortality related to ARI in Pakistan in the past decade, the country still ranks high in pneumonia-associated deaths. Therefore, a better understanding of risk factors in rural regions of Pakistan is necessitated to design appropriate preventive measures that can be coupled with improved case management. To understand the epidemiology of the disease in rural areas, we conducted a survey to identify risk factors associated with ARI among under-five children in rural Jamshoro, Pakistan.

## Methods

### Study Design, Sampling Procedure, and Selection of Sample

This study is a retrospective case–control design, drawing data from a baseline assessment conducted prior to a cluster randomized controlled trial (protocol under review). The retrospective case–control study was conducted between October and December 2018 in 10 union councils (UCs) in Taluka Kotri, Jamshoro District. Jamshoro is one of the 29 districts of the Sindh province, located about 150 km from the provincial capital, Karachi. Jamshoro consists of four Talukas/Tehsils including Kotri. Taluka Kotri is two-thirds urban, with an estimated population of 0.43 million and 64,500 children under 5 years of age. It consists of 12 primary health care facilities and one tertiary and one district headquarter hospitals. Acute respiratory infections cases are more common during the winter season in Pakistan (13).

### Study Population

The study population were children between 0 and 23 months of age within the 10 UCs. Children were excluded if they had missing immunization or breastfeeding information. A case was defined as a child who had fast breathing or difficulty in breathing due to a problem in the chest only or both the chest and nose in the 2 weeks prior to the survey. A control was defined as a child between 0 and 23 months of age who did not have fast or difficulty breathing 2 weeks prior to the survey. Two controls were matched with a case based on age in months.

### Measures and Outcomes

Acute respiratory infections was assessed using a structured questionnaire, which asked caretakers of children if the child had fever or an illness with cough that resulted in shorter, faster, or rapid breathing and if the child had fast or difficulty breathing due to a problem in the chest or due to a blocked or runny nose. As a follow-up, chest indrawing due to fever and cough was also asked. Factors associated with ARI included gender and age of the child, mothers' age and education, number of people living in a room, breastfeeding status, wealth index, WASH indicators (improved sources of water and sanitation), and immunization status of the child. A summary of independent outcome definitions is presented in Table 1.

**Table 1:** Measures for socioeconomic and WASH outcomes.

	**Overcrowding**	**Defined as six or more people living in one room**
Sociodemographic variables	Wealth index	A composite score was created as a measure of socioeconomic status based on a range of consumer goods owned by households (television, refrigerator, mobile phone, internet, bed, etc.), housing characteristics (source of drinking water, type of sanitation facilities, flooring material, access to electricity, number of rooms used for sleeping, fuel used for cooking), and ownership of assets, land, and livestock. Wealth scores were generated according to these indicators and surveyed households were ranked according to their wealth score.
Behavioral and WASH practices	Solid fuel	Includes LPG, natural gas, biogas, coal, and wood—fuels that provide heating through combustion
	Improved source of drinking water	Includes piped water, public taps, tube wells, boreholes, protected dug wells, springs, rainwater, and bottled water
	Improved source of water for sewage	Includes piped water, public taps, tube wells, boreholes, protected dug wells, springs, and rainwater
	Improved source of sanitation	Includes non-shared toilets that are flush/pour toilets to piped sewer systems, septic tanks, and pit latrines

### Data Collection

Households with at least one under 2-year-old child registered with the Lady Health Worker (LHW) Program by the Ministry of Health were visited for the study. Female community health workers (CHWs) accompanied the LHWs to prepare a list of households visited by the LHW to map the catchment area covered by each LHW. A total of 29,258 households were enlisted in the 10 UCs identified for the study, covered by 210 LHWs. Out of this, 6,657 households had at least one child between 0 and 23 months of age. Convenience sampling was conducted, where households were selected according to the ease of the CHW to visit. All children between 0 and 23 months of age with symptoms of ARI who resided within the catchment area were included in the study as cases. Controls also resided within the LHW Program catchment area.

A structured questionnaire was administered to the parents/caregivers of the child by the CHW. The questionnaire contained questions about the socioeconomic and demographic status of the household and the health and nutrition status of the child. The questionnaire is available on request. Data was collected on paper-based questionnaires, was rechecked and verified for accuracy, and then double entered to avoid transcription errors.

### Sample Size and Statistical Analysis

Between October and December 2018, a total of 1,071 cases and 2,142 matched controls were recruited to the study. This gives a 90% power for detecting an odds ratio (OR) of ≥1.5 as significant at the 5% level if the prevalence of exposure among controls is 10–90% and correlation between cases and control is 0.2–1.

Analyses were undertaken using STATA version 15. Exploratory analyses were summarized with frequencies and proportions. The predictors of ARI were determined by conditional logistic regression. We initially performed bivariate analyses to examine the association between each risk factor and the outcome variable (Tables 2, 3). A multivariable model was adjusted for all risk factors significant at *p* < 0.2 in bivariate model (model A). Another multivariable model was fitted by including variables with *p* < 0.2 in the bivariate model (model B) using a backward elimination method, and variables with *p* < 0.05 were retained within the model. Results were reported as matched OR and their 95% confidence intervals (CIs). Plausible interaction and multi-collinearity were additionally assessed. A *p*-value of <0.05 was deemed statistically significant.

**Table 2:** Descriptive and univariate analysis of demographic and socioeconomic characteristics of households associated with ARI.

**Variables**	**Category**	**Cases**	**Controls**	**Odds ratio**	***P* > |*z*|**
		***N* = 1,071**	***N* = 2,142**		
Gender	Male	586 (54.7%)	1,047 (48.9%)	Ref	Ref
	Female	485 (45.3%)	1,095 (51.1%)	0.79 (0.68–0.92)	0.002
Number of people per room	≤3	366 (34.2%)	705 (65.8%)	Ref	Ref
	More than 3	722 (33.7%)	1,420 (66.3%)	0.98 (0.84–1.14)	0.792
Mother's age	≤20 year	80 (7.5%)	133 (6.2%)	Ref	Ref
	20–30	652 (61.0%)	1,299 (60.8%)	0.83 (0.62–1.12)	0.218
	>30	337 (31.5%)	703 (32.9%)	0.80 (0.59–1.08)	0.145
Mother's education	No education	558 (52.2%)	1,139 (53.3%)	Ref	Ref
	Primary	178 (16.7%)	313 (14.7%)	1.16 (0.94–1.43)	0.173
	Secondary	204 (19.1%)	385 (18.0%)	1.08 (0.88–1.32)	0.450
	Higher	129 (12.1%)	298 (14.0%)	0.88 (0.69–1.11)	0.275
Father's education	No education	338 (31.9%)	633 (29.8%)	Ref	Ref
	Primary	170 (16.0%)	348 (16.4%)	0.91 (0.72–1.14)	0.393
	Secondary	313 (29.5%)	588 (27.6%)	0.99 (0.82–1.19)	0.871
	Higher	240 (22.6%)	558 (26.2%)	0.80 (0.65–0.98)	0.028
Wealth index (quintiles)	Poorest	224 (20.9%)	419 (19.6%)	Ref	Ref
	Poor	221 (20.6%)	422 (19.7%)	0.98 (0.78–1.23)	0.873
	Middle	223 (20.8%)	419 (19.6%)	1.00 (0.79–1.26)	0.971
	Rich	205 (19.1%)	439 (20.5%)	0.87 (0.69–1.10)	0.236
	Richest	198 (18.5%)	443 (20.7%)	0.84 (0.66–1.06)	0.132

*Row percentages comparing cases to controls*.

**Table 3:** Descriptive and univariate analysis of household behavioral and WASH practices, and health and nutrition of participants with ARI.

**Variables**	**Categories**	**Cases**	**Controls**	**Odds ratio**	***P* > |*z*|**
		***N* = 1,071**	***N* = 2,142**		
Cooking done	Indoor	1,036 (96.7%)	2,084 (97.3%)	Ref	Ref
	Outdoor	35 (3.3%)	58 (2.7%)	1.22 (0.79–1.87)	0.369
Solid fuel	No	858 (80.1%)	1,709 (79.8%)	Ref	Ref
	Yes	213 (19.9%)	433 (20.2%)	0.98 (0.82–1.18)	0.828
Improved source of drinking water	No	234 (21.8%)	442 (20.6%)	Ref	Ref
	Yes	837 (78.2%)	1,700 (79.4%)	0.93 (0.78–1.11)	0.424
Improved source of water for usage	No	243 (22.7%)	446 (20.8%)	Ref	Ref
	Yes	828 (77.3%)	1,696 (79.2%)	0.90 (0.75–1.07)	0.222
Improved source of sanitation	No	847 (79.1%)	1,742 (81.3%)	Ref	Ref
	Yes	224 (20.9%)	400 (18.7%)	1.16 (0.96–1.39)	0.126
Make water safer to drink?	No	520 (48.6%)	1,005 (46.9%)	Ref	Ref
	Yes	551 (51.4%)	1,137 (53.1%)	0.94 (0.81–1.09)	0.378
Wash hands before feeding infant	No	806 (75.3%)	1,637 (76.4%)	Ref	Ref
	Yes	265 (24.7%)	505 (23.6%)	1.07 (0.90–1.27)	0.464
Colostrum given to baby	No	110 (10.3%)	202 (9.4%)	Ref	Ref
	Yes	961 (89.7%)	1,940 (90.6%)	0.91 (0.71–1.16)	0.448
Currently breastfeeding	No	232 (21.7%)	428 (20.0%)	Ref	Ref
	Yes	839 (78.3%)	1,714 (80.0%)	0.90 (0.74–1.08)	0.251
Exclusively breastfed (0–6 months)	No	827 (77.2%)	1,569 (73.2%)	Ref	Ref
	Yes	244 (22.8%)	573 (26.8%)	0.81 (0.69–0.96)	0.017
Immunizations	No	468 (43.7%)	905 (42.3%)	Ref	Ref
	Yes	603 (56.3%)	1237 (57.8%)	0.94 (0.81–1.09)	0.436
Comorbid with diarrhea	No	637 (59.5%)	1516 (70.8%)	Ref	Ref
	Yes	434 (40.5%)	626 (29.2%)	1.64 (1.41–1.91)	<0.001

### Ethics Statement

An informed and signed consent was given by caretakers of the children included in the study. In the situation where the caretaker was illiterate, consent was signed by thumb impression in the presence of a witness. If the household was locked, the family was not available, or the caretaker refused informed consent, the next household with an eligible child was selected. Ethical approval was obtained from the Ethics Review Committee of the Aga Khan University (4722-Ped-ERC-17).

## Results

We identified 1,071 cases with symptoms of ARI 2 weeks prior to the survey, who were matched with 2,142 controls. Table 2 presents the demographic and socioeconomic characteristics associated with ARI through a univariate logistic regression analysis. Risk factors associated with a lower likelihood to develop ARI included the female gender (OR: 0.79, 95% CI: 0.68–0.92) and children with fathers who achieved higher education compared to no education (OR: 0.80, 95% CI: 0.65–0.98). Moreover, children who were exclusively breastfed were less likely to get ARI (OR: 0.81, 95% CI: 0.69–0.96), and children comorbid with diarrhea were more likely to get ARI (OR: 1.64, 95% CI: 1.41–1.91) (Table 3).

We conducted a multivariable model fit to adjust variables associated with the incidence of ARI (Table 4). The female gender (OR: 0.78, 95% CI: 0.67–0.91) and exclusive breastfeeding from 0 to 6 months (OR: 0.81, 95% CI: 0.69–0.97) were protective against ARI, whereas infants comorbid with diarrhea had a higher likelihood of ARI (OR: 1.64, 95% CI: 1.40–1.91).

**Table 4:** Multivariable analysis (fully adjusted and reduced) for risk factors associated with ARI.

		**Fully adjusted (model A)**		**Reduced (model B)**	
**Variables**	**Categories**	**amOR^†^**	**P>|z|**	**amOR^†^**	***P* > |*z*|**
Gender	Male	Ref	Ref	Ref	Ref
	Female	0.79 (0.68–0.91)	0.002	0.78 (0.67–0.91)	0.002
Mother age	≤20 year	Ref	Ref		
	20–30	0.82 (0.61–1.10)	0.180		
	>30	0.821 (0.60–1.12)	0.218		
Mother's education	No education	Ref	Ref		
	Primary	1.200 (0.96–1.50)	0.112		
	Secondary	1.123 (0.90–1.41)	0.312		
	Higher	1.015 (0.76–1.35)	0.917		
Father's education	No education	Ref			
	Primary	0.922 (0.73–1.17)			
	Secondary	1.009 (0.82–1.24)			
	Higher	0.869 (0.68–1.11)			
Exclusively breastfed (0–6 months)	No	Ref	Ref	Ref	Ref
	Yes	0.821 (0.69–0.98)	0.026	0.81 (0.69–0.97)	0.019
Wealth index (quintiles)	Poorest	Ref	Ref		
	Poor	0.964 (0.76–1.22)	0.761		
	Middle	0.983 (0.77–1.26)	0.894		
	Rich	0.900 (0.69–1.17)	0.438		
	Richest	0.911 (0.68–1.22)	0.527		
Improved source of sanitation	No	Ref	Ref		
	Yes	1.158 (0.96–1.40)	0.132		
Comorbid with diarrhea	No	Ref	Ref	Ref	Ref
	Yes	1.579 (1.35–1.85)	<0.001	1.64 (1.40–1.91)	<0.001

*A—Model includes all predictors significant at p < 0.2 in bivariate analysis*.

*B—Parsimonious model selected using backward elimination, p < 0.05 considered significant*.

† *Adjusted matched odds ratio*.

## Discussion

Our study evaluated the risk factors associated with ARI in a rural district in Sindh, Pakistan among children between 0 and 23 months of age by comparing cases to controls who did not experience symptoms of ARI. Our study found that female infants were less likely to develop ARI symptoms as opposed to male infants. A study in Pakistan identified that male children were more affected by ARI than female children, but the finding was not significant (8). Similar findings to our study have been identified in literature from neighboring countries, Bangladesh and India, where cultural and social norms are similar to those in our study context (14–17). This could be due to cultural norms where male infants have a higher exposure to the outdoors, smoke, and air pollution, whereas females tend to stay indoors from a young age and spend more time with their mothers.

We identified that exclusive breastfeeding was protective against ARI. Breastfeeding provides children with natural immunity and has shown to be protective against several diseases. A meta-analysis of 10 studies from developing and developed countries on risk factors of acute lower respiratory infections (ALRI) identified that infants who were not exclusively breastfed were more than twice as likely to develop severe ALRI than those who were exclusively breastfed (18). Partial or no breastfeeding has shown to increase the risk of ARI-related mortality by 2.40 in a verbal autopsy study (19). Another study in India identified that timely initiation of breastfeeding resulted in a lower occurrence of ARI (17). Moreover, a Nigerian study highlighted the importance of protective immunoglobulins in breastmilk and the significance of breastfeeding in preventing malnutrition, which in turn was protective against ARI (20). Similarly, diarrhea presents as a risk factor to ARI due to the association between diarrhea, malnutrition, and compromised immunity in infants, therefore resulting in a higher risk of contracting ARI. A quantitative analysis among Indian and Nepali children found an increased incidence of ARI when diarrhea occurred within 28 days prior to the onset of ARI, with an increased comorbidity with an increase in diarrhea severity (21). Encouraging the uptake of exclusive breastfeeding has the potential to trickle down its benefits on not only reducing ARI in children but also reducing the incidence of diarrhea, which can have a synergetic effect in reducing ARI in the developing world.

Paternal education was associated with ARI in the univariate analysis, but was not significant in the final multivariable model. This was also identified in a study in urban slums of Gulbarga, India, where children belonging to illiterate fathers were twice as likely to develop ARI than those with literate fathers (16). A similar study in Nigeria also found that children whose parents have poor parental education had thrice the risk of developing pneumonia (20). The impact of paternal education could be due to the cultural practice that exists in Pakistan, where fathers are deemed decision makers in households and influence health-seeking behaviors, nutrition, and general living conditions of the family, which directly impact childhood morbidities (22, 23). A recently conducted study in Pakistan concluded that education of caretakers can improve the recognition of danger signs in children and consequently improve care-seeking practices (24).

Contrary to other studies, our study did not identify socioeconomic, WASH-related, or household characteristics as risk factors of ARI (8, 11, 14, 17, 25, 26). This could be because our study was conducted in one locality of Jamshoro District. The wealth index of the participants is subjective and only comparable to the wealth index of the study sample and is therefore not generalizable to the whole population. It is likely that our study population did not have stark differences between wealth quintiles, since they belonged to a similar socioeconomic background, so we could not identify any association between wealth index and ARI. Nevertheless, access to healthcare to address an illness is difficult in rural areas of the country due to distances, lack of healthcare facilities, and transportation, which reduces healthcare utilization in rural areas (27). This ascertains the need for interventions targeted in rural areas where children have delayed treatment to their illness.

Although there has been a sharp decline of respiratory infection burden across the globe, including a nearly 50% decline in Pakistan over the past two decades, the country still faces the highest number of pneumonia and diarrheal deaths globally (6). As our study indicates, diarrhea in children is a common risk factor for ARI; therefore, targeting interventions to reduce diarrhea in children can interchangeably impact the morbidities from ARI reduction. Diarrhea and pneumonia are also risk factors for undernutrition and malnutrition in children; therefore, it is imperative to not only reduce the incidence of ARI in children to prevent mortality and morbidity related to ARI but to also diminish the outcomes of ARI and the long-term consequences presented with ARI at a young age (28, 29).

Using a matched case–control study design, our study evaluated numerous determinant factors of ARI, but failed to take into account smoking within the household and nutritional status of the child, both of which have been identified as imperative risk factors to ARI in literature. A limitation to our study was that all our participants were from one locality, so it could not identify large socioeconomic differences between participants; thus, our study is not generalizable to entirely rural or urban settings within the country. Another limitation is that, since we used questionnaires to recall incidence of ARI through symptoms, recall bias may have affected the accuracy of data. Moreover, we did not confirm the status of ARI and did not perform clinical examinations to diagnose the infants. Another limitation to our study was that our age group was restricted to children under 2 years of age and would likely not apply to the under-five age group.

## Conclusion

Pakistan is one of the most susceptible regions to ARI and ARI-related mortalities in children. Despite significant improvement, health outcomes still have a long way to go. Our study highlights risk factors associated with ARI in children between 0 and 23 months, with female children and those exclusively breastfed having a lower likelihood for ARI and diarrhea as a common risk factor for the disease. Improving factors associated with diarrhea, education, and awareness to prevent ARI and promoting breastfeeding have the potential to effectively reduce ARI among children. Furthermore, studying the interaction between ARI, diarrhea, breastfeeding, and undernutrition can direct programs to take a multifaceted approach to tackling under-five mortality and morbidity in Pakistan.

## Data Availability Statement

The raw data supporting the conclusions of this article will be made available by the authors, without undue reservation.

## Ethics Statement

The studies involving human participants were reviewed and approved by the Ethics Review Committee of the Aga Khan University (4722-Ped-ERC-17). The patients/participants provided their written informed consent to participate in this study.

## Author Contributions

ZB, SS, FM, and SA conceived and designed the study and reviewed final manuscript. SC and AN developed the tools, conducted the trainings, and implemented the study. MJ, AHu, and AR cleaned the data and conducted the final analysis. FM, SC, MB, AN, MU, ZM, AHa, SA, SS, and ZB contributed to the development of manuscript. All authors contributed to the article and approved the submitted version.

## Funding

This study was funded by the Bill & Melinda Gates Foundation (BMGF) through grant OPP1148892.

## Author Disclaimer

Findings and conclusions have been demonstrated by authors and do not represent the funding agency.

## Conflict of Interest

The authors declare that the research was conducted in the absence of any commercial or financial relationships that could be construed as a potential conflict of interest.

## Publisher's Note

All claims expressed in this article are solely those of the authors and do not necessarily represent those of their affiliated organizations, or those of the publisher, the editors and the reviewers. Any product that may be evaluated in this article, or claim that may be made by its manufacturer, is not guaranteed or endorsed by the publisher.
